# Strengthening English language undergraduates’ presentation skills: A blackboard-mediated intervention program

**DOI:** 10.1371/journal.pone.0289936

**Published:** 2023-08-23

**Authors:** Sami Algouzi, Ali Abbas Falah Alzubi, Mohd Nazim

**Affiliations:** 1 Associate Professor of Linguistics, Department of English, College of Languages and Translation, Najran University, Najran, Saudi Arabia; 2 Assistant Professor of Applied Linguistics, Department of English, College of Languages and Translation, Najran University, Najran, Saudi Arabia; 3 Associate Professor, English Department, College of Languages and Translation, Najran University, Najran, Saudi Arabia; The University of Auckland, NEW ZEALAND

## Abstract

Studies and reports indicate that some graduates struggle to find jobs, in part because they lack the key presentation skills and competencies the labor market needs. Thus, this research investigated the effectiveness of a Blackboard-mediated intervention program in strengthening English as a foreign language (EFL) students’ presentation skills. The research followed a quasi-experimental (time series) design, delivering workshops on presentation skills and collecting data from the students (N = 30) using a set of instruments: a pre-and post-assessment checklist and semi-structured interviews. The results showed that the students’ presentation skills improved significantly post intervention. Also, the participants reported positive attitudes concerning the intervention. Drawing on these findings, recommendations and suggestions are presented.

## Introduction

Presentation skills refer to the communicative abilities a person must possess to deliver engaging, informative, educational, enlightening, and attractive content, such as enthusiasm, a focus on the audience, keeping things simple, and excellent body language. Tursunoy describes oral presentations as a significant component of the English as a foreign language (EFL) classroom today in various parts of the world [1]. As Evans and Morrison point out, presentations are now frequently used as assessment tools or as class exercises in all academic fields, especially in English as a second language (ESL) and EFL settings [2]. Moreover, Yang notes that the EFL context has emerged as essential for fostering oral competence in environments that are less favorable in terms of oral socialization [3].

In studies of communication, presentation skills have attracted the attention of researchers. For example, Kim [4] and Evans [5] argue that presentation skills are considered successful communicative goals. Presentation skills are widely required in today’s professional world and are essential for graduates, who need to acquire these skills to present on diverse occasions and at various events. Graduates understand the importance of presentation skills such as those highlighted by Dung, who states that the presenter needs a professional appearance, proper pronunciation, and fluency to engage the audience, and they also understand that practice can boost the oral and communicative aspects of the presentation [6]. However, they find acquiring and using these skills challenging for various reasons. In this regard, drawing on the views of learners, Osterman suggests that the development of oral skills should begin with practicing communication [7].

In this research, we argue that presentation skills are a necessary consideration with reference to the Saudi Vision 2030 and labor market needs, and competence in oral presentation should be a subject of prominence. Competence in oral presentation comprises the knowledge, skills, and attitudes required to speak in public, where the goals may include informing or persuading the audience, or self-expression [8]. Oral presentation skills are considered key for employability [9], and communication, especially in the oral mode, has been identified as one of the essential skills for the 21st century. Presentation skills bring students benefits, such as lifelong learning skills. Moreover, the learning experience can help them develop appropriate skills if they are trained professionally. Presentation skills give learners an effective means of bridging the gap between language study and language use. Making presentations requires students to use all four skills in a natural, integrative way [10]. These days, university students and graduates are typically required to have the ability to make presentations in English to a public audience [11]. Oral presentation is not only part of 21st-century skills but is also required when students enter the workplace [12]. Therefore, higher education courses commonly integrate oral presentations as part of the course activities and/or learning objectives [13].

Presentation-related studies have been widely researched. Previous research has focused on the impact of oral presentation on language proficiency, speaking ability, oral communication abilities, self-confidence, attitudes, challenges, and factors of influence in learning presentation skills [8, 14–18]. However, to the best of our knowledge, no research thus far has been conducted on the use of online instructional interventions to train students in how to present themselves in English. This study, undertaken at Najran University, addressed many aspects of oral presentations, such as organization, content, language, style, and delivery, as well as students’ lack of enthusiasm.

It was expected that this study would lead to a significant improvement in undergraduates’ presentation abilities, which are vital in today’s professional world and to meet labor market expectations. The rising need for graduates with effective presentation skills requires more effective, innovative, and result-oriented instruction. Better teaching and learning methodologies are needed to enhance students’ presentation skills and teachers must pay special attention to this aspect of learning. The intervention in this study highlighted crucial areas in presentation abilities that many researchers may not have consider. Therefore, this study aimed to promote undergraduates’ presentation skills, consistent with the goals of Saudi Vision 2030 and labor market needs, via a Blackboard-mediated intervention program. The findings provide crucial suggestions about making presentations and advance proposals concerning the essential elements for an effective presentation.

### Theoretical framework

Bandura’s social cognitive theory contends that human actions are influenced by personal, behavioral, and environmental factors [19]. According to this theory, seeing others in social interactions, one’s experiences, and outside media influences might contribute to an individual’s knowledge acquisition and behavior [20], as people acquire behaviors and cognitive techniques through watching how others behave [21]. When individuals observe activity being modelled and the consequences of that conduct, they remember the sequence of events and use this knowledge to influence future behavior [22]. In this process, the environment, behavior, and cognition all play important roles in shaping growth in reciprocal triadic interactions [19, 20].

Thus, the foundation of social cognitive theory is a process of information acquisition or learning directly related to model observation. According to Zhou and Brown [20], three factors contribute to model observation: model characteristics, such as high status, competence, and power; observer attributes, such as talent and courage, confidence, self-esteem, and independence; and model action consequences, such as self-efficacy and self-regulation. Effective modeling provides broad norms and techniques for coping with various circumstances. This can be provided through interpersonal imitation or media sources [19].

### Review of the literature

The available literature suggests an increasing focus among researchers on the importance of presentation skills and studying the challenges learners face in presenting. Some of the main challenges learners face in making presentations are background knowledge, anxiety, motivation, language, grammar, vocabulary, and pronunciation [23–29]. This is perhaps not surprising as many studies have found that presenting is a multi-layered and challenging task. Morreale points out that presenting requires considerable preparation, for example organizing content, incorporating relevant information and ideas, and selecting the appropriate attire [30]. It is necessary for students to combat these challenges since mastering slide shows, demonstrations, lectures, or speeches can assist presenters communicate with audiences by utilizing words and images [31].

Examining the presentations of a group of TESOL graduates, Zareva showed that the students acted in a variety of identity roles: guiding the audience through the information, recounting their research and decision-making processes, drawing attention to how the information was organized, and clarifying the purpose of their presentation and the structure of their argument [32]. Finding that students had difficulties providing presentation content for audiences, Melvina and Dona Alicia argued that teachers should spend more time introducing them to the broad skills they need when giving presentations [33].

Numerous factors influence presentation skills, including the ability to speak in English, which is something students are often afraid of doing [34]. Rumiyati and Seftika observe that speaking in front of a crowd is one of the most difficult tasks for EFL students [35]. Tsang identified a significant correlation between students’ perceived competence regarding the delivery of oral presentations and their level of anxiety concerning public speaking [36]. Similarly, Waluyo and Rofiah found that students’ performance in presentations is predicted by situational and potential confidence and communication confidence [16].

Background knowledge, psychology, language and style, preparation, and the instructor are some of the key factors that influence learners’ delivery of presentations [37–41]. Indriani found that qualities such as eye contact, body posture, and voice were further characteristics that aided pre-service teachers’ English-speaking abilities [42]. Among these, Worawong et al. identified hand gestures were the strategy most used by students in their oral presentations [43]. Okada et al. [44] and Yano [45] showed that self-monitoring, peer evaluation, and model observation have positive effects on improving learners’ oral presentation skills.

Technology can significantly enhance the general standard of one’s presentation in various ways. However, students’ readiness to embrace such technology and focus during presentations is critical. As a basis, Donohoe observed that presenters commonly utilize PowerPoint in the modern era to transmit information or media via slides as the medium offers adaptable presentation styles [46]. However, it is important to note that the development of information technologies has paved the way for new means of making presentations. Many technologies are available, such as Prezi, Keynote, and PowerPoint, as well as a range of venues, such as blogs, Facebook, and YouTube [47–49]. Thus, students should be encouraged to deliver their presentations by exploring different technologies, which can lead to better oral communication skills compared to traditional presentation tools [50].

Alshobramy found that applying social learning theory increased the speaking ability of secondary school EFL students naturally by providing innovative and adaptable learning experiences [51]. Fauzi showed that a multimedia-based presentation approach assisted students in developing their speaking and presentation skills, as well as their confidence [52]. Mahdi also reported that multimedia devices had a positive impact on the development of presentation and speaking skills among students [8]. Salem reported that TED lectures enhanced business students’ oral presentation abilities and vocabulary uptake/retention levels [53]. Also, the students were more enthusiastic, motivated, and eager to produce outstanding presentations as they grew more self-assured and relaxed. Sirisrimangkorn revealed that project-based learning using presentations had significant effects on students’ speaking skills [54]. Burhanuddin claimed that the individual presentation method was effective in enhancing students’ confidence and providing them with the experience of speaking in front of a crowd [14]. The results also indicated that the task gave them more awareness and self-evaluation on how to perform good public speaking. Hida examined the effectiveness of collaborative learning in co-constructing knowledge and skills in giving oral presentations in English classrooms in Japan and found that the learners primarily acquired five benefits: noticing the gap, knowledge co-construction, overcoming weakness, behavior modeling, and psychological improvement [55]. Pham et al. conducted a study aiming at measuring English-majored students’ views of their speaking skills, especially presentation skills. The results showed that most students were not confident about their presentation skills because of fears of making mistakes in vocabulary usage and grammar, lack of fluency, and so on [17].

There are very few studies on employing ICT-mediated programs to improve the presentation skills of EFL learners. However, some studies have suggested that learners experience difficulties in terms of anxiety, learning issues, and media access and use. For example, Solmaz employed Pecha Kucha to develop EFL learners’ speaking and oral presentation skills. Thematic analysis not only highlighted the advantages of the program, such as developing speaking and presentation skills, enhancing self-confidence, and improving time management, but also drawbacks, such as increased anxiety, a steep learning curve, and format constraints [56]. Among other studies examining the integration of technology in oral presentations [57–60], some found that this can pose difficulties in terms of the students’ language competence. Some students believed that the time given to them was insufficient, while others considered that their poor speaking abilities were to blame for their difficulties in presenting. Students also experienced fear of speaking since they understood that virtual audiences would view recordings of their oral presentations later.

To summarize, previous studies, both with and without the integration of technology, have investigated presentations with a focus on numerous different aspects. A review of the literature suggests that existing research on strengthening EFL learners’ presentation skills is very fragmented, lacks theoretical grounding and has received little empirical attention with particular reference to implementing an intervention. This research was premised on the belief that implementing an intervention program could enhance EFL learners’ presentation skills, making them better qualified for the labor market. The study investigated how a multilayered intervention program delivered through a series of workshops on Blackboard might help EFL students become successful presenters.

The study entailed designing and implementing a Blackboard-mediated interventional program aimed at improving undergraduates’ presentation skills in terms of organization, content, communication, delivery, and enthusiasm. The study utilized Blackboard as a platform to present the intervention as many presentations, and indeed job interviews, take place online, particularly since COVID-19. Other reasons for choosing Blackboard as a platform concerned convenience for the students in terms of time, place, effort, reference, and cost. The study sought to address the following research questions:

1. What impact does a Blackboard-mediated intervention program have on EFL undergraduates’ presentation skills?
2. What are the participants’ views of the experience of the Blackboard-mediated intervention program and its effect on their presentation skills?

## Methodology

### Research design and context

The research adopted a quasi-experimental design to achieve the study objectives. This study aimed to investigate how effectively a Blackboard-mediated intervention program would be in strengthening EFL students’ presentation skills. An assessment checklist and semi-structured interviews were used to collect the data from undergraduates at the College of Languages and Translation at Najran University in the Kingdom of the Saudi Arabia in the second semester of the academic year 2023.

### Population and sample

The study population comprised undergraduates majoring in the English language and translation programs at Najran University in 2023. The study sample was based on purposive sampling and students’ voluntary participation. Those students who agreed to participate in the study completed two copies of the written informed consent form; they kept one copy and returned the other to the researchers. The Ethical Approval Committee at the Deanship of Scientific Research, Najran University granted approval to conduct the study [009773-021280-DS]. It should be noted that the researchers had no access to personal information that could identify individual participants at any time during or after data collection.

The study sample comprised two groups, 30 students in total, recruited to the study in the second semester of 2023. All the participants were Saudi, aged 22–23 years, and enrolled in the 9th and 10th levels of two courses: Contrastive Linguistics and Drama. They had been exposed to English language instruction for 11 years at school and university and all spoke Arabic as their mother tongue. They were studying EFL in a formal context and their English level should be considered upper-intermediate. Thus, they should have been able to initiate presentations, raise inquiries, and express their opinions about what they were studying in relation to the instructional material.

### Study instruments

The study applied two instruments for data collection: a pre-and post-assessment checklist and semi-structured interview. The researchers designed the assessment checklist with reference to presentation assessment rubrics available online, such as one developed by Owen Williamson at the University of Texas (https://utminers.utep.edu/omwilliamson/engl1311/Presrubric.doc) and another developed by the Justice Institute of British Columbia (https://www.jibc.ca/sites/default/files/library/files/Group_Presentation_Marking_Rubric.doc). The assessment checklist included presentation skills (25 items) distributed across five main domains: organization, content, communication, delivery, and enthusiasm. Each dimension contained five items.

*Organization* included aspects such as defining the background and importance of the topic, stating objectives that can identify relevant questions, presenting information in a logical sequence, summarizing the major points of the presentation, and providing attendees with a “take-home” message. *Content* included gaining the attention of the audience, defining technical terms, including relevant material, preparedness of the content, and presenting an obvious conclusion. *Communication* included good language skills and pronunciation, demonstrating good grammar and choice of words, using rhythm, intonation, accent, and tone variation, effective pace of delivery, being fluent and articulate, and using no fillers (umm, like), or long pauses. *Delivery* included items about maintaining good eye contact with the audience, using gestures in addition to a clear and audible voice, using well-prepared informative handouts, notes, and visual aids, presenting within the assigned time limits, and answering questions professionally. Finally, *enthusiasm* contained items about demonstrating strong enthusiasm throughout the presentation, increasing audience understanding and knowledge of the topic, convincing the audience to recognize the validity and importance of the subject drawing on evidence, being relaxed and confident with no/minimal hesitation throughout the talk, and being in professional attire.

Before the treatment program, the participants were asked to present topics related to two subjects they were studying (Contrastive Linguistics and Drama), and their performance was assessed using the checklist. Then, they were trained in presentation skills by one of the teachers with experience in this area. After that, they were again asked to present the topics related to their subjects and assessed using the same checklist.

Semi-structured interviews were employed in which the participants were asked about their experience of learning presentation skills, their attitudes, and suggestions for further improvements. The participants were interviewed immediately after the post-assessment by another teacher who had not conducted the intervention. The interviews were estimated to last 8–10 minutes. They were conducted in an office in the Department of English and audio-recorded. The semi-structured interview questions were as follows:

1. How would describe your experience of the presentation skills workshops?
2. What new presentation skills did you learn in the workshops?
3. How did you feel after taking the presentation skills workshops?
4. What things did you like/ dislike about the presentation skills workshops?
5. Do you have any suggestions for making the presentation skills workshops more fruitful? Please elaborate.

#### Validity and reliability

A jury of five experts checked both instruments, the assessment checklist and the interview questions, to establish content validity. The experts were specialized in English language teaching and technology-based learning and teaching and had more than 10 years of experience in teaching and assessment. The experts had the study tools and objectives to verify that the tools could produce valid data to answer the research questions. They also checked the applicability of the items in the Saudi context. Finally, they suggested working on language issues.

To establish the internal consistency of the assessment checklist, the researchers applied Pearson’s correlation coefficient (r) to check the relationship between items and the checklist as a whole. The checklist was applied to assess the performance of a sample of 20 students who did not participate in the study. Table 1 shows the results of the correlation.

**Table 1:** Pearson’s correlation coefficients for the assessment checklist.

Item	r	Item	r	Item	r	Item	r	Item	r
1	.604**	6	.652**	11	.598**	16	.769**	21	.654**
2	.616**	7	.505*	12	.590**	17	.579**	22	.611**
3	.745**	8	.789**	13	.647**	18	.524*	23	.630**
4	.524*	9	.673**	14	.579**	19	.616**	24	.634**
5	.654**	10	.597**	15	.504*	20	.504*	25	.543*

** Significant at p = 0.01

* Significant at p = 0.05.

As shown in Table 1, the values of the Pearson correlation coefficients for the relation between each item and the whole scale ranged between 0.505 and 0.769 and were all significant at p = 0.01 or p = 0.05, demonstrating the validity of the checklist.

To verify the reliability of the assessment checklist, two assessors evaluated the performance of the exploratory sample (N = 20). The two assessors were faculty members in the Department of English, specializing in English language teaching and assessment. They had been teaching English for more than 15 years. The authors oriented them on the study topic, objectives, and evaluation checklist (dimensions and items). Any points they did not understand were clarified. The assessors were instructed to use a separate checklist for each student and to conduct the evaluation while the student was presenting. The reliability of the assessment checklist was calculated based on the level of agreement between the assessors (inter-rater reliability): Level of agreement/(no. of agreements + no. of disagreements) [13]. Table 2 presents the results.

**Table 2:** Reliability of the assessment checklist.

Item	Agreement	Disagreement	Reliability coefficient %
**Domain 1: Organization**	88	12	88
Defines background and importance of topic	18	2	90
States objective and can identify relevant questions	17	3	85
Presents information in a logical sequence	19	1	95
Summarizes major points of talk	16	4	80
Provides attendees with a “take-home” message	18	2	90
**Domain 2: Content**	87	13	87
Introduction is attention-grabbing	17	3	85
Defines technical terms in a language comprehensible for the target audience	16	4	80
Includes relevant material that contains useful information	19	1	95
Content is well-prepared and points reflect their relative importance	16	4	80
Presents a clear conclusion that summarizes the presentation	19	1	95
**Domain 3: Communication**	86	14	86
Uses good language skills and pronunciation	17	3	85
Demonstrates good grammar and choice of words	16	4	80
Uses rhythm, intonation, accent, and variation of tone with an effective pace of delivery	18	2	90
Is fluent and articulate	16	4	80
Uses no fillers (umm, like), long pauses, etc.	19	1	95
**Domain 4: Delivery**	90	10	90
Maintains good eye contact with the audience	19	1	95
Uses body language appropriately in addition to a clear and audible voice	18	2	90
Uses well prepared informative/not distracting handouts/notes/visual aids	19	1	95
Presentation is within the assigned time limits	16	4	80
Answers questions professionally	18	2	90
**Domain 5: Enthusiasm**	87	13	87
Demonstrates strong enthusiasm about topic during entire presentation	19	1	95
Significantly increases audience understanding and knowledge of topic	17	3	85
Convinces audience to recognize the validity and importance of the subject	18	2	90
Is relaxed and confident with no/minimal hesitation throughout talk	16	4	80
Attire is professional (business casual or formal)	17	3	85
**Overall**	**438**	**62**	**87.6**

Table 2 shows that the assessment checklist was reliable (87.6%). The reliability coefficient values of domains ranged between 86% and 90%.

### Instructional intervention

The study drew on social cognitive theory as a theoretical foundation to create and implement a Blackboard-mediated intervention aimed at improving undergraduates’ oral presentation skills. A variety of factors influenced the selection of this theoretical framework. First and foremost, the researchers aimed to draw as much as possible from the existing literature on the procedures used in the current study to improve EFL students’ oral presentation skills. This research is aligned with Solmaz [56] in considering “the long-term character of the development process of oral presentation skills, described as central professional skills” (p.16). Moreover, the purpose of the study corresponds to Bandura’s view that social cognitive theory is particularly well adapted to explaining the evolution of complex behavior, such as oral presentation skills [19]. Based on this theory, the researchers considered three main factors that contribute to changing behavior—personal, behavioral, and environmental—in that people learn new knowledge by watching others and use it in the future to change their behaviors. In addition, the study utilized previous research, such as the work of Zareva [32], who referred to the roles TESOL graduates played when examining presentations, such as guiding the audience through the information, recounting their research and decision-making processes, drawing attention to how the information was organized, and clarifying the purpose of their presentation and the structure of their argument.

The study provided a training program on presentation skills through workshops in which the participants watched how others presented, learned from the process, and applied it in the future to change their behavior. Interventions in the educational sphere provide students with the required or desired assistance they need in the form of capabilities, competencies, skills, etc., which could not be obtained or developed during an educational program and the lack of which may adversely impact graduates’ future or career opportunities. De Grez observed that “to design an instructional intervention, we have to be clear about its objectives. We have to determine the outcomes of the intervention focusing on the acquisition and development of oral presentation skills” [61, p.57]. This study aimed to design and carry out a Blackboard-mediated intervention program, conducting workshops to strengthen EFL undergraduates’ presentation skills in line with the goals of Saudi Vision 2030 and labor market needs.

The program focused on enhancing the 30 participants’ knowledge of how to make their presentations effective and improving their performance. The content was divided into five workshops, which introduced the principles of presentation to EFL students and trained them how to present themselves well. The participants were told that presentations are synonymous with demonstrations, lectures, or speeches. They were also made aware that presentations are tailored to persuade, inspire, motivate, or present a new idea/concept to people termed “the audience” who are at the core of any presentation. After the orientation session on the concept, the researchers introduced themselves and the study.

The participants delivered a presentation before the intervention. Both the participants and the researchers were able to identify weaknesses in the organization, content, language, style, and delivery, as well as a lack of enthusiasm on the part of the presenters. Following the initial presentation, the participants were asked to participate in the Blackboard-mediated workshops. Each session lasted one hour, with the last 10 minutes devoted to questions and answers. The researchers also wanted the participants to learn through observation, so they shared relevant videos about the five specific areas crucial in presentations with a focus on the “do’s and don’ts.”

The research procedure consisted of three phases. In the first phase, the participants were required to give presentations and their performance was evaluated using the assessment checklist elaborated by the researchers. This identified issues with organization, content, language, communication style, delivery, and enthusiasm. The second phase comprised the series of five workshops, conducted on Blackboard by an experienced trainer, to instruct the students in how to present effectively and professionally.

The first workshop concerned the organization of presentations, highlighting the need for a clear beginning, middle, and end. The trainer pointed out that the presenter needs to organize ideas logically throughout the presentation and follow the order in a very organized fashion, striving for clear transitions between individual points, slides, and topics. Moreover, the presentation needs to be structured based on the audience and purpose. In addition, the trainer highlighted other key points, such as defining the background and importance of the topic, stating objectives that can identify relevant questions, presenting information in a logical sequence, summarizing the main points of the presentation, and providing attendees with a “take-home” message. The trainer shared videos (https://www.youtube.com/watch?v=4bwDr7WVBwo) on presentation organization. After watching the video clips, the participants were invited to have a discussion, followed by a question-and-answer session.

The second workshop concerned the substance of presentations in terms of the content. The trainer emphasized the need for unique and important ideas and information. The presenter must use reputable and pertinent sources and cite those sources when necessary. Information must be concise and pertinent to the audience. Again, the trainer addressed several crucial aspects related to content, including gaining the attention of the audience, defining technical terms, incorporating relevant material, preparing the content well, and presenting an obvious conclusion. The trainer shared videos related to content (https://www.youtube.com/watch?v=Yl_FJAOcFgQ) and instructed the participants to pay close attention to considerations of significance and originality.

The third workshop sought to underscore that word choice can make aspects of the presentation clear and memorable if selected well. The trainer highlighted that language, style, and communication are significantly impacted by word choice. The session addressed denotative and connotative concepts, referring to presenting the message clearly, expressing ideas effectively, and choosing respectful and unbiased language. The trainer highlighted several key points, such as the language of presentations typically being somewhat less formal than academic writing, the need to present the main points one by one and pause at the end of each main point to give the audience time to absorb the information and take notes and using phrases to indicate moving on to a new point. In addition, one should consider several aspects under the theme of language, style, and communication, for example, using good language skills and pronunciation, demonstrating good grammar and choice of words, using rhythm, intonation, accent, tone variation, and an effective pace of delivery, being fluent and articulate, and using no fillers (umm, like), or long pauses, etc. The trainer then shared clips on communication, style, and language (https://www.youtube.com/watch?v=ewVCnfMGnFY), demonstrating that word choice and language use are crucial for communication.

The fourth workshop concerned delivery and addressed a range of factors, from body language and word choice to vocal variety. The trainer highlighted that a good presenter has a passion for the subject and can convey—and perhaps elicit—that emotion in the audience. The workshop stressed the need to make a connection with the audience through eye contact, facial expressions, gestures, and/or vocal tone, as well as to avoid fillers (e.g., umm) and hesitations. These all contribute to communicating the presenter’s professionalism and confidence, inviting audience engagement. In addition, the session covered providing well-prepared, informative handouts, notes, and visual aids, presenting within the assigned time limits, and answering questions professionally. The trainer then shared videos on delivery (https://www.youtube.com/watch?v=S5c1susCPAE&t=8s).

The last workshop concerned the need for the presenter to show enthusiasm and covered aspects such as using a wide range of gestures (especially when presenting to a large audience on stage), making eye contact with attendees, and speaking with a smile and energy. Thus, the session emphasized the role of body language and facial expressions, as well as highlighting that the presenter’s clothing should not draw attention. Linking back to previous sessions, the workshop noted the relevance of enthusiasm in conveying knowledge of the topic and convincing the audience of the validity and importance of the subject by being relaxed and confident. Again, the trainer shared videos on this aspect of presenting (https://www.youtube.com/watch?v=5naThX63pF0).

In the third phase, students were required to give a presentation and their performance was again assessed using the same checklist as previously. After the presentation, a researcher interviewed the students, asking questions related to their experience of engaging in the presentation skills workshops, their attitudes and feelings about the intervention, and their suggestions for improvement.

### Data analysis

The Statistical Package for the Social Sciences (SPSS) v. 25 was used to analyze the data collected from the pre-and post-assessment checklist. To establish the effectiveness of the training program in enhancing the participants’ presentation skills, the study employed paired sample t-tests. The researchers conducted content analysis of the qualitative data from the semi-structured interviews, based on repeated occurrences and grouped under main themes.

## Results

### The effect of the training program on students’ presentation skills

Table 3 presents the results of the impact of the intervention program on students’ presentation skills, drawing on the pre- and post-assessment for the individual domains and whole scale.

**Table 3:** Paired samples t-test (pre- & post-assessment).

Domain		N	M	S.D.	t	df	Sig. (2-tailed)	Effect size d	Level of effect
**Organization**	Pre	30	1.77	.521	16.337	29	.000	2.97	Large
	Post	30	4.60	.578					
**Content**	Pre	30	2.07	.785	14.653	29	.000	2.66	Large
	Post	30	4.65	.559					
**Communication**	Pre	30	2.38	.751	12.526	29	.000	2.28	Large
	Post	30	4.50	.455					
**Delivery**	Pre	30	2.10	.649	13.964	29	.000	2.54	Large
	Post	30	4.50	.670					
**Enthusiasm**	Pre	30	2.10	.960	15.577	29	.000	2.83	Large
	Post	30	4.97	.183					
**Total**	Pre	30		2.75	19.863	29	.000	3.61	Large
	Post	30		1.72					

Table 3 shows significant differences at the level of 0.05 before and after the training program in favor of the post-performance (t(29) = 19.863, p > .05). This result indicates that the training program was highly effective in improving the students’ presentation skills.

### Students’ reflections on the presentation skills program

Several key themes emerged from the semi-structured interviews with the students concerning their experience of the presentation skills workshops. For most of the interviewees, it was their first time presenting. They reported that the training program was a helpful, interesting, and exciting experience, and they benefited a great deal from it. It helped rid them of anxiety and fear, and they started to feel more confident. In addition, they learned that they should be well-prepared and not appear confused. Furthermore, the training program assisted them in improving their presentation skills in terms of facing the audience and delivering the topic as required. The students also reported that they benefited from the feedback from peers. The following are some of the interviewees’ responses to the first question in the interview concerning their experience of the presentation skills workshops:

S1. “It is very interesting and helpful. It is the first time I’ve done a presentation.”
S20. “It was a very good experience.”
S12. “A wonderful experience that developed my speaking skills and improved my way of meeting the audience and conveying the idea to them in the required form.”
S13. “A beautiful experience to enhance self-confidence and break the barrier of public fear.”
S10. “I learned not to get confused during the presentation and come prepared.”

### Presentation skills learned

The interviewees’ responses concerning the presentation skills they acquired through the workshops revealed that they learned to speak in front of the public with confidence, to interact with the listeners and ask questions, to raise their voices to attract attention, to pay attention to their body language and tone of voice, to talk without reference to the book, and to give and take examples from other students. Also, they learned how to explain and present without becoming stressed, to present without inappropriate interruption, and to be fluent and accurate. They broke the barrier of fear and stress and improved their self-confidence. The following are some excerpts from the interviewees’ answers:

S3. “Speaking skills, looking around the listeners, asking questions for them to interact with, and raising the voice to attract attention.”
S11. “The eye contact and the hand signals, and the importance of preparing for the presentation.”
S13. “Fluency and accuracy in speaking and interacting with the public.”
S16. “Facing the audience, increasing self-confidence, and exploring skills about communicating information in its simplest form.”
S19. “Speaking skills without confusion, the skill of explaining and communicating information.”

### Feelings about the experience of engaging in the workshops

The students also described their feelings after taking the presentation skills workshops. They were excited and felt positive about the experience. They were very happy to be trained in presentation skills and to achieve something significant. They broke the barrier and the tension and were proud of what they had achieved. They gained a high level of confidence and morale. These aspects are evidenced in the following excerpts:

S2. “Awesome and broke the stress barrier.”
S3. “I feel a sense of accomplishment after I took this step for the first time. Great feeling and development of diction skills and help later.”
S6. “Nice and I felt the sense of teaching.”
S7. “It’s a nice feeling and I see myself developing in speaking.”
S13. “Feeling excited and happy to gain the skill of recitation.”
S18. “I feel that I have gained a high level of confidence and morale.”

### Likes and dislikes

Students reflected on the things they liked or disliked about the presentation skills workshops. They liked the interaction with their peers, strengthening and refining their speaking skills, the seriousness of the sessions, meeting with others, skills development, reviewing errors, the organization of the workshop, enthusiasm, fun, facing the public, and peer support. They also liked the idea of using technology, such as laptops and data presentations. On the negative side, two students were rather tense and confused, which they reported led to some errors during their presentations. The following excerpts provide evidential support for the emergent themes:

S3. “The things that I liked is that strengthening and refining diction skills. The things I didn’t like were the tension just before the presentation.”
S7. “Everything I liked and most specially, it increased my self-confidence by speaking.”
S11. “The things I like is the experience and some confidence make me would like to do it again and thing I do not like is during the presentation I got confused and I said something wrong.”
S18. “I liked during my presentation the interaction of my student friends.”
S19. “I liked that it was enthusiastic and fun, and the interaction between classmates.”

### Suggestions for improving the presentation skills workshops

The students were asked for suggestions to make the presentation skills workshops more fruitful. They recommended repeating the workshops because of the benefits they provided. Also, some students suggested including presentations as part of their assessment in various subjects. These points are illustrated in the following excerpts:

S5. “More of these shows to develop students’ skills.”
S12. “More of these workshops because it is of great benefit to the student.”
S15. “We hope that the distinguished doctors include this participation in all subjects and integrate it into monthly grades.”
S17. “I hope this beautiful event continues.”
S19. “I suggest that this offer be weekly in order to benefit more.”

## Discussion

This research investigated the impact of a training program mediated by the Blackboard platform on improving EFL students’ presentation skills. Based on the results, the students who engaged in the intervention attained significant improvements in their scores for their presentation skills post-treatment compared to pre-treatment in all five domains: organization, content, communication, delivery, and enthusiasm. This indicates the effectiveness of the intervention.

Several factors may have contributed to this result, such as the integration of the Blackboard platform, enhanced motivation, reduced anxiety, stress, and tension, and the students’ recognition of the need to improve their speaking and presentation skills. The integration of Blackboard contributed to the effectiveness of the program as it is user-friendly, free, and accessible to users, regardless of place and time. Moreover, the training sessions were recorded and the students could refer to them at any time. In addition, the students were motivated to participate and engage due to their need to improve their presentation skills, as evidenced in the interviews. The analysis of the interviewees’ responses revealed that they found the intervention program a very good means of refining their presentation skills. They enjoyed the experience and reported it assisted them in facing their fear of speaking in front of the public and improving their body language, speaking skills, and self-confidence. In addition, they learned to interact with the audience and attract attention.

The results of this research are consistent with previous studies. Similar to this intervention, research has found that presentation qualities like eye contact, body posture, and voice aid English-speaking abilities [42], and project-based learning using presentation can significantly affect students’ speaking skills [55], with students’ oral presentation skills improving significantly after instruction due to enhanced confidence and the experience of speaking in front of a crowd [14]. As in this study, previous research has reported participants favoring a multimedia design [15], which improves students’ confidence [52], and also collaborative learning, as it enables the co-construction of knowledge and skills [56]. Such courses can enhance students’ oral presentation abilities and vocabulary uptake/retention levels [54], as well as making them more enthusiastic, motivated, and eager to produce outstanding presentations as they grow more self-assured and relaxed. In addition, these results are consistent with Brooks [61], who showed that oral presentation allows learners to use their second language to communicate with others naturally. De Grez [62] also suggested that students are highly motivated to learn how to present. In terms of the use of technology, this study employed Blackboard to facilitate deliver of the intervention program, which may have helped improve students’ performance [15]. This result accords with previous studies that used technology to improve students’ speaking and presentation skills, employing a multimedia approach [51–57].

The results of this study also support the claim of social cognitive theory that learners require exposure and practice to enhance their acquisition of skills that will help them in their future careers. In this research, the participants observed how others (trainer and peers) behaved, stored this knowledge, and used it to change their behavior when presenting post-intervention. Thus, learners can refine their behavior based on observation and experience. The training program allowed the participants room for exposure and practice in presenting themselves properly. They learned how to organize their presentations, engage the audience, and deliver content effectively, as well as to present with enthusiasm.

According to Bandura [63], “man’s capacity to learn by observation enables him to acquire large, integrated units of behavior by example without having to build up the pattern gradually by tedious trial and error” (p. 2). Alshobramy argues that the application of social learning theory can naturally increase speaking ability by providing innovative and adaptable learning experiences [52]. Hence, consistent with theory, this study supports the goals of the Saudi Vision 2030 and the labor market needs of skilled graduates in enabling them to design and deliver effective presentations.

## Conclusion

This research focused on enhancing undergraduates’ (life-long) presentation skills through a Blackboard-mediated intervention program. In contrast to prior research that employed ICT-mediated programs to develop presentation skills and found EFL learners experienced difficulties in terms of anxiety, learning issues, language competence, and media access [55, 57–60], the results of this study showed that the learners’ levels of fear, learning problems, and access issues decreased during the intervention. Also, the program proved highly effective in improving the EFL participants’ presentation skills, and their attitudes and feedback were positive. Therefore, the study contributes to the existing body of knowledge by presenting evidence of the value of utilizing technology, specifically Blackboard, in a planned program to improve students’ presentation skills, which are in great demand in the labor market.

Students who master English will have an added advantage if they possess presentation skills and their job opportunities will be greater. Accordingly, this study argues the need to include presentation skills as part of students’ course assessment. In addition, technology can play a role in enhancing students’ presentation skills; they can utilize technology to record themselves and to review their mistakes, and thus improve their performance.

This research has certain limitations, most notably the participants’ gender; all the participants were male due to the gender-based segregation in Saudi higher education. Moreover, the relatively small number of participants means the findings are not generalizable. In this regard, similar studies could be undertaken in different contexts employing the same interventional program and tools—or similar—and enable the comparison of results. In Saudi Arabia, given the effectiveness of the intervention in this study, it is recommended that stakeholders conduct more workshops on presentation skills, as they support the goals of the Saudi Vision 2030 and address the needs of the labor market. Further research and pedagogical practice could consider a range of methods, such as peer and self-assessment, to measure students’ acquisition of presentation skills. Finally, more research is needed to focus on comparing students’ competence and performance in presentation skills.
